# Investigating Emotion Perception via the Two-Dimensional Affect and Feeling Space: An Example of a Cross-Cultural Study Among Chinese and Non-Chinese Participants

**DOI:** 10.3389/fpsyg.2021.662610

**Published:** 2021-07-23

**Authors:** Pernelle Lorette

**Affiliations:** Department of English Studies, University of Mannheim, Mannheim, Germany

**Keywords:** emotion perception, affect, self-report, instrument, cross-cultural, emotion measurement

## Abstract

The categorical approach to cross-cultural emotion perception research has mainly relied on constrained experimental tasks, which have arguably biased previous findings and attenuated cross-cultural differences. On the other hand, in the constructionist approach, conclusions on the universal nature of valence and arousal have mainly been indirectly drawn based on participants' word-matching or free-sorting behaviors, but studies based on participants' continuous valence and arousal ratings are very scarce. When it comes to self-reports of specific emotion perception, constructionists tend to rely on free labeling, which has its own limitations. In an attempt to move beyond the limitations of previous methods, a new instrument called the Two-Dimensional Affect and Feeling Space (2DAFS) has been developed. The 2DAFS is a useful, innovative, and user-friendly instrument that can easily be integrated in online surveys and allows for the collection of both continuous valence and arousal ratings and categorical emotion perception data in a quick and flexible way. In order to illustrate the usefulness of this tool, a cross-cultural emotion perception study based on the 2DAFS is reported. The results indicate the cross-cultural variation in valence and arousal perception, suggesting that the minimal universality hypothesis might need to be more nuanced.

## Introduction

Despite the long history and multidisciplinarity of emotion research, the nature of emotion is still very much debated (Fox, 2018; Berent et al., 2020). Not only between but also within disciplines, differences in conceptualizations of emotions have led to different methodologies to investigate emotions, hence to different results and conclusions. Focusing particularly on the fields of linguistics and psychology, which are the most relevant to the present contribution, emotions have been approached in various ways. Whereas, the basic paradigm has mainly endorsed a categorical approach to emotions, the constructionist paradigm supports the dimensional approach to emotion, highlighting the fuzziness of borders between different emotion categories and the heterogeneity of experiences and expressions within such “constructed” categories (e.g., Quigley and Barrett, 2014). In this contribution, an emotion is regarded as a construction of the mind based on exteroceptive and interoceptive sensations—i.e., perceptions of the environment and of internal physiological states, respectively, —and the meaning one attributes to these sensations (e.g., Russell, 2003; Barrett, 2006, 2017a). According to this view, the most reliable way to know which emotion(s) an individual is experiencing—i.e., is perceiving in themselves—or is perceiving in someone else is to ask the individual themselves about their own perception (Scherer et al., 2013; Barrett, 2017a). Thus, in this approach, a valid instrument enabling the collection of such self-report data is crucial. As will be argued in the next section, the currently available instruments present drawbacks. In an attempt to overcome the limitations of previous studies (see e.g., Barrett et al., 2019), a new instrument called the Two-Dimensional Affect and Feeling Space (2DAFS) has been developed, which is particularly advantageous for survey studies. The main aim of this article is to present the 2DAFS and demonstrate its usefulness for emotion research.

In the next section, categorical and dimensional research approaches will be presented in more detail, before reviewing several emotion perception studies based on self-reports—either self-reports of own experienced emotions or those of perceived emotions experienced by someone else. Particular attention will be drawn onto the methodological choices made in these studies and their potential repercussions on the research outcomes. Next, the 2DAFS will be introduced. To illustrate the usefulness of this instrument, a study of a cross-cultural emotion perception based on the 2DAFS will be presented. The aim of this study is to investigate how emotions expressed by a Mandarin speaker are perceived by Chinese participants and by participants who are unfamiliar with the Chinese language and culture. Thus, the instrument will be described in the methodology section, together with the design and the participants of this study. The result section will discuss the results of this illustrative study, while the general discussion of this article will be dedicated at the evaluation of the instrument and its potential contribution to the field of emotion research.

## Different Conceptualizations of Emotion

The theoretical spectrum on the nature of emotion can be thought of as a continuum ranging from more categorical accounts of emotion, such as the basic emotion theory (BET), to more dimensional accounts, such as the psychological constructionist approach (Gross and Barrett, 2011). Whereas, the categorical approach regards emotions as discrete, well-defined, and rather homogeneous entities, the constructionist approach emphasizes the heterogeneity and the fuzziness of the boundaries characterizing emotion categories. The BET strongly supports the view of an emotion as a discrete entity triggered automatically by a stimulus in the environment, and occasioning a set of specific physiological and behavioral reactions (e.g., Tomkins, 1962, 1963; Izard, 1971; Ekman, 1992; Keltner and Shiota, 2003), such as facial movements, vocal modulations, and activation of the peripheral nervous system. Traditionally, BET has posited the existence of six basic emotions, namely, happiness, surprise, fear, disgust, anger, and sadness. These clearly defined emotion categories are assumed to be universally experienced in the same way, and hence universally recognizable, be it from their facial or vocal manifestations. Similarly to BET, appraisal theory postulates the existence of discrete emotion categories, arising from a (usually external) trigger, which brings about a chain of specific reactions. However, whereas in BET an emotion is assumed to occur as a reflex once it is triggered by an external event, appraisal theory posits an essential role of the emotion experiencer, as an emotion will only arise once the experiencer has imparted meaning to the stimulus, based on their needs, goals, and values (e.g., Arnold, 1960; Scherer, 2001). In short, just as BET, appraisal theory implies the idea of a one-to-one link between emotion and response, actually mediated by a one-to-one link between emotion and appraisal and a one-to-one link between appraisal and response (van Reekum et al., 2004). However, those appraisals are conceptualized as dimensions, along which corresponding emotion categories can be identified (Wundt, 1897; Scherer, 1984). Further removed from BET on this theoretical continuum are constructionist approaches, such as the theory of constructed emotions (Barrett, 2017b), which regards emotions as individual constructions of the mind based on (the continuous dimensions of) how pleasant one is feeling—i.e., valence—and how activated one is feeling—i.e., arousal. Such an approach refutes any kind of one-to-one link between emotion and its manifestations. This implies that an emotion can be interpreted, but is not a perceiver-independent object in the physical world that can be recognized (Barrett, 2017a). Consequently, a dimensional aspect permeates the emotion perception research of both appraisal scholars and constructionists—be it directly in their data collection (e.g., Scherer et al., 2013) or in their data analysis (e.g., Russell, 1980; Gendron et al., 2014b)—while the basic paradigm is more categorically oriented.

Constructionists strongly criticize the assumption that basic emotions are universally experienced and expressed in the same way. According to constructionists (e.g., Russell, 1995, 2015; Nelson and Russell, 2013; Gendron et al., 2014b, 2018; Barrett et al., 2019), the so-called universality thesis (Nelson and Russell, 2013) is based on methodological choices that bias results and attenuate cross-cultural differences. The early investigations into emotion perception were conducted in the categorical approach by Paul Ekman and his team and typically implemented a forced-choice response format. This format forced participants to indicate that they have perceived (one of the few predetermined) emotions, while they may in fact have perceived a purely physiological phenomenon or an action (e.g., Gendron et al., 2014b). These seminal studies focused on the cross-cultural recognition of basic emotions based on facial cues. Participants were usually presented with (static) photographs of an actor displaying different (prototypical) facial expressions. People from different cultures demonstrated similar choice patterns when asked to choose one of the six emotion labels corresponding to the emotion displayed on the actor's face (e.g., Ekman et al., 1969; Ekman and Friesen, 1971; Ekman, 1972). However, studies conducted in the constructionist approach, which have implemented less-constrained tasks, have found much weaker support for the universality thesis than studies conducted in the categorical approach. In those studies, various data collection approaches were used, such as free-sorting (Gendron et al., 2014b), free-labeling (Crivelli et al., 2017; Gendron et al., 2020), word-matching (Crivelli et al., 2016a), and choice-from-array (Gendron et al., 2020) tasks. Overall, constructionist studies reveal that when forced to choose a label corresponding to the emotion displayed in a stimulus, Westerners' response patterns conform more with the patterns expected by the universality thesis than non-Westerners' response patterns, demonstrating the non-universality of this thesis (Crivelli et al., 2016a). Moreover, once participants can freely label the perceived emotion, even Westerners' rates of agreement with the intended emotions are lower than the rates reported in forced-choice studies, demonstrating the biases introduced by constrained tasks (Gendron et al., 2014a). It has also been demonstrated that facial configurations (Gendron et al., 2014b; Crivelli et al., 2016b) or vocalizations (Gendron et al., 2014a) that are normatively associated with specific emotions in Western societies—such as the gasping face for fear—are not always associated with the same emotion in non-Western samples, or are even associated with mere behaviors or actions rather than with any mental states. Thus, these studies do not reveal cross-cultural perception of specific emotions—i.e., do not support the universality thesis. The response patterns do, however, provide support for the cross-cultural stability of valence and arousal perception, since positivity/negativity and activation/inactivation are perceived similarly across cultures (Crivelli et al., 2016a, 2017). This supports Russell's (1995, 2003) minimal universality hypothesis, which claims that valence and arousal are the only universal aspects of emotions. Strikingly, few studies supporting the minimal universality hypothesis are based on direct ratings of perceived valence and arousal level by participants from different cultures. One exception is Crivelli's et al. (2017) study, which found similar valence and arousal perception across cultures. However, these valence and arousal ratings were based on dichotomous judgments—i.e., categorized as either pleasant or unpleasant and either aroused or calm emotional states, which might prevent the discovery of more subtle differences in perception of valence and arousal between cultural groups. When asking participants to rate valence and arousal, taking the continuous nature of valence and arousal into account might lead to different conclusions, but only a handful of studies have followed that path so far. Sneddon et al. (2011) presented low-pass-filtered visual–vocal recordings of an Irish person to participants from Northern Ireland, Serbia, Peru, and Guatemala. Participants had to rate the strength of positive and negative emotions *via* a continuous slider. Note that the use of this single slider made valence and intensity ratings indistinct from one another. Participants agreed on the overall positive or negative character of the valence in each clip, but slight differences appeared in the extent to which participants from different countries thought the person in the recordings was feeling pleasant. Similarly, Koeda et al. (2013) directly collected continuous intensity, valence and arousal ratings of Japanese and Canadian participants hearing non-verbal vocalizations expressed by Canadian actors. Each rating was collected *via* a scale ranging from 0 to 100. More extreme levels of valence—i.e., higher for positive emotions and lower for negative emotions—were perceived by Canadian participants in half of the stimuli. Regarding arousal, only one difference was revealed for the stimulus conveying sadness, with higher arousal perceptions by the Japanese group. Thus, although more research is needed to confirm these first hints, these findings provide the first indications that the minimal universality hypothesis might need to be reformulated in a more refined way.

The previous paragraphs have described how different conceptualizations of emotions have led to different methodologies to investigate emotion perception, implementing more or less constrained tasks. In an attempt to move away from constraining forced-choice response formats, Russell et al. (1989) developed a first instrument, called the Affect Grid, to assess descriptive or subjective judgments of valence and arousal. The Affect Grid is a 9x9-grid defined vertically by a nine-level scale representing arousal—with “extremely high arousal” on the top and “extreme sleepiness” on the bottom—and horizontally by a nine-level scale representing valence—with “extremely unpleasant feelings” on the left and “extremely pleasant feelings” on the right. The center of the grid represents “a neutral, average, everyday feeling. It is neither positive nor negative” (Russell et al., 1989, p. 501). Judgments are indicated by drawing a cross in one of the squares of the grid according to the defining dimensions. This instrument is easy to use and rather simple in design. However, with today's technologies, people are more used to using continuous sliders rather than placing a cross in a grid. Furthermore, Russell et al.'s (1989) instrument contains additional emotion labels placed at the periphery of the grid to guide participants' report of their valence and arousal perception. However, this might bias participants' perception of valence and arousal, as emotion labels do not have a universal meaning, but the conceptual representation of a term is bound with cultural and individual variation (Osgood et al., 1975; Fontaine et al., 2013). Moreover, this instrument is solely restricted to the collection of valence and arousal ratings and does not allow for data collection pertaining to emotion categorization.

**Figure 1:**
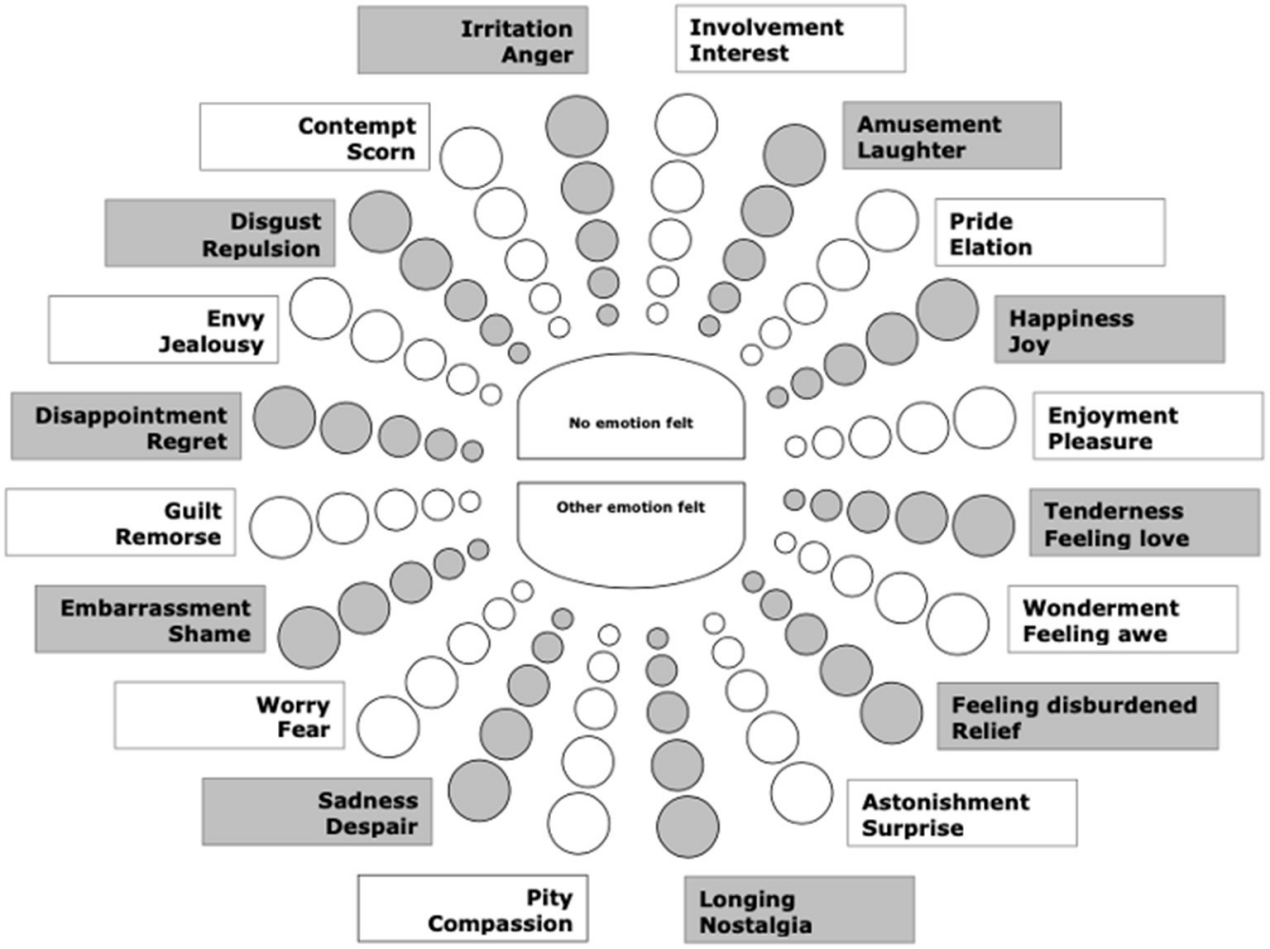
The Geneva Emotion Wheel (GEW, reproduced with permission from Scherer, 2005; Scherer et al., 2013).

Inspired by the Affect Grid and Russell's circumplex model of affect (see e.g., Russell, 1980), Scherer et al. (2013) developed the Geneva Emotion Wheel (GEW) to collect self-report data combining a dimensional and a categorical approach (see Figure 1). In the GEW, 20 labels for the so-called *emotion family* are graphically arranged along the circumference of a circular two-dimensional space defined by the horizontal dimension of valence and the vertical dimension of control/power. Different emotion labels within each emotion family are placed within the circle—they appear when the mouse goes over their position—with the least intense emotion labels of the family placed close to the center of the circle, and other emotion labels being placed increasingly far from the center of the circle as they are assumed to refer to more intense emotions from that family. The center of the circle refers to “no emotion felt” or “other emotion felt.” An important advantage of this user-friendly instrument is that it combines a dimensional and a categorical approach and enables emotion categorization self-report *via* a less-constrained task than the ones implemented in most previous research. However, an emotion label is attached to each dimensional rating, which arguably also biases the dimensional ratings. Moreover, three dimensions are combined in a two-dimensional space, namely, valence, intensity, and control/power—with control/power appearing to be rather abstract and difficult to rate for participants. Researchers interested in the perception of arousal thus have to turn to another instrument, as intensity and arousal are related but distinct concepts, which are not linearly correlated with each other (Kuppens et al., 2013)—e.g., one can feel intensely depressed but with very low arousal.

Finally, other attempts at moving away from constrained forced-choice response formats with few alternatives have been proposed implementing pictograms or emojis. The Self-Assessment Manikin (SAM, Bradley and Lang, 1994), for instance, enables the (successive) collection of valence, arousal, and dominance ratings *via* three individual 9-point Likert-type scales defined horizontally by five pictograms and the four spaces between them. The “pleasure” scale ranges from a frowning character to a smiling character. The arousal scale ranges from a neutral character whose mouth is a straight line to a character with a depicted explosion in his upper body. The dominance scale ranges from a small character in size to a big character in size. Although this instrument has been extensively used and allows for quick data collection, reports of participants questioning or misinterpreting the meaning of these pictograms are also very common (e.g., Broekens and Brinkman, 2013; Chen et al., 2018), suggesting that it is not intuitive to use and requires much explanation. This is unsurprising since these pictograms were designed three decades ago, well-before the extensive use of emojis, GIFs, and the like in everyday (virtual) conversations. Moreover, assimilating a stereotypical facial expression (or physiological state) with an emotional dimension—e.g., low valence with frowning and high valence with smiling—is problematic (Barrett, 2017a) as it suggests neglect of the high variability of behaviors and experiences associated with low or high valence, arousal, and dominance. The same arguments apply to instruments implementing emojis (e.g., Betella and Verschure, 2016; Toet et al., 2018), as the prototypical facial expressions and emotional states can be (mis)leading for the participant to rate affective dimensions. Another major drawback of the use of emojis is that their interpretation is culture-specific (e.g., Takahashi et al., 2017; Guntuku et al., 2019) and thus problematic for cross-cultural studies.

In summary, findings have so far not univocally supported the universality thesis nor the minimal universality hypothesis. Although the basic paradigm defends the universality thesis, their findings might be biased by the restrictive forced-choice response format typically implemented in their study. On the other hand, constructionists endorse the minimal universality hypothesis, but the bulk of the evidence supporting this hypothesis is based on either indirect inferences, or response patterns, or dichotomous judgments, rather than on valence and arousal being directly rated in a continuous way by participants from different cultures. In order to fill this gap, the current study aims at investigating whether the minimal universality hypothesis, which postulates that valence and arousal are universal, still holds when Chinese participants and participants unfamiliar with the Chinese language and culture continuously rate the valence and arousal level of a Chinese speaker in dynamic visual recordings. The research question of this study is the following:

Is the valence and arousal level of a Mandarin speaker's internal state perceived similarly by Chinese and non-Chinese participants in visual recordings?

## Materials and Methods

### Instrument: The Two-Dimensional Affect and Feeling Space

In order to overcome the shortcomings of instruments used in previous studies, the 2DAFS was developed. The 2DAFS allows to collect both self-reports of continuous valence-and-arousal ratings and self-reports of categorical perceptions in a fast and user-friendly way and can easily be embedded in online surveys.

**Figure 2:**
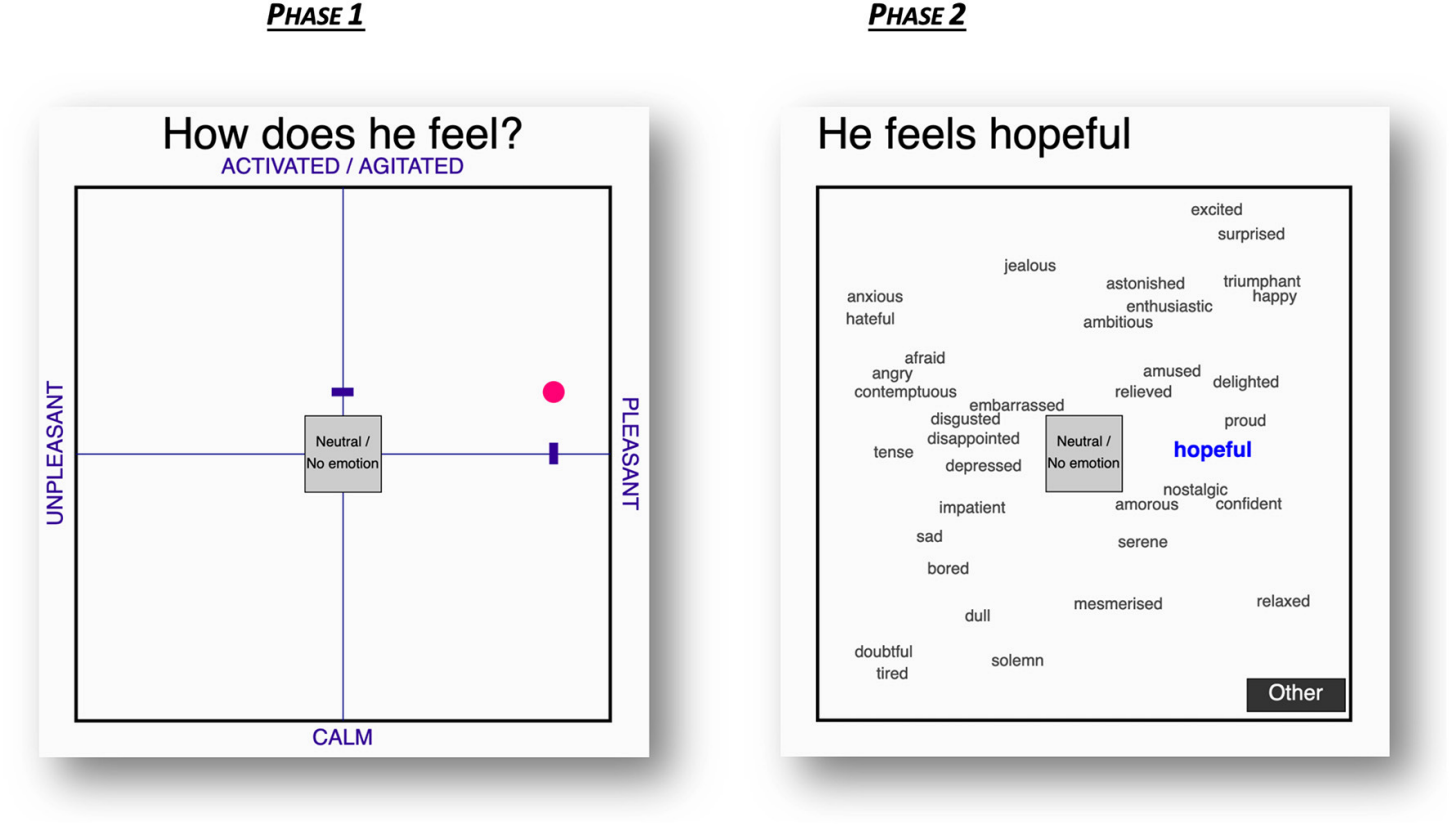
First and second phases of the English version of the response format.

The software implementing the interactive response format has been developed with p5.js, a JavaScript library, which is based on the principles of the programming language processing. The response format is structured in two phases, with the screen of phase 2 replacing the original screen once participants have completed phase 1 (see Figure 2). The first phase corresponds to the rating of the arousal and valence level of the perceived emotional state of the person depicted in the stimulus. A two-dimensional space appears, characterized by a horizontal axis x labeled “unpleasant” at the left and “pleasant” at the right extremity and a vertical axis y labeled “calm” at the bottom and “activated/agitated” at the top extremity. The participant's cursor can move around in the space, and the projection of its position on both dimensions is highlighted with an interactive pointer on each axis—moving simultaneously with, and accordingly to, the participant's cursor. In order to maximize the clarity and the enjoyable character of the instrument, the axes and their anchoring labels are printed in purple and the cursor is highlighted with a pink dot. The question “How does he feel?”—referring to the speaker in the stimulus—is displayed above the space. The participant has to click on a spot in the space depending on the perceived “pleasantness”—i.e., valence—and “agitation”—i.e., arousal—of the emotional state of the actor. Hence, the coordinates of the chosen spot correspond to the perceived valence (x) and arousal (y) level of the emotional state of the actor. The more pleasant the speaker is feeling, the more the pink dot should be placed on the right side of the space. Concurrently, the more agitated the speaker is feeling, the more the pink dot should be placed in the upper part of the space. In case the participant does not perceive any valence or arousal, they have the possibility to click in the center of the space labeled “neutral/no emotion.” Once the participant has clicked on a spot in the space, the pink dot unties from the participant's cursor and gets fixed on this spot. The participant can either click a second time on the pink dot to confirm their choice, or click somewhere else in the space to choose another spot if they want to correct their choice. The coordinates of this spot are then stored, enabling statistical analyses of these values. Valence and arousal ratings can both range from 0 (extremely low valence and extremely low arousal, respectively), to 800 (extremely high valence and extremely high arousal, respectively). As the delimitation of the “neutral area” in the two-dimensional space ranges from coordinates 365 to 435, variation in the coordinates of any click within this range is meaningless. Therefore, ratings with a value between 365 and 435 are recoded to 400 to eliminate meaningless variation in the data. Moreover, differences in ratings that are smaller than 60 are regarded as meaningless and thus negligible. This threshold corresponds to the smallest adjustment in location of the cursor in the two-dimensional space that a participant can indicate by shifting their pointer and which they can perceive as located at a significantly different spot on the axis, even on a small device. This threshold was determined by several tests conducted by the researcher and the software developer on different mobile devices to see the smallest distance that can be indicated by moving the cursor with a finger on a small touchscreen. As the scale is adaptive, this means that participants using a bigger screen might have been able to wittingly indicate finer-grained differences by shifting the position of their cursor. However, 60 was chosen as a safe threshold to limit the risk of type I errors—although this inevitably increases the risk of type II errors. A pilot study using 6-point Likert-type scales ranging from “completely disagree” to “completely agree” to test the user-friendliness of the 2DAFS among six first-language speakers and 32 second-language speakers of English indicated that participants found this two-dimensional space clear and intuitive to use (“I find it clear/intuitive to know how to rate the level of pleasantness and agitation with this tool,” mean = 4.5, *SD* = 0.9).

Once the participant has confirmed the spot of the pink dot, the screen moves on to phase 2. The axes inside the space disappear together with the four labels at their extremities and 36 labels appear in the space. The incomplete sentence “He feels …” is displayed above the space, inviting the participant to choose one of the 36 labels which could fit in this sentence. The 36 labels included in the instrument are anxious, jealous, hateful, afraid, angry, contemptuous, embarrassed, disgusted, disappointed, tense, depressed, impatient, sad, bored, dull, doubtful, tired, solemn, mesmerized, relaxed, serene, amorous, confident, nostalgic, hopeful, proud, relieved, delighted, amused, ambitious, enthusiastic, astonished, happy, triumphant, surprised, and excited. All labels correspond to adjectives that describe how someone is feeling and they can all fit in the sentence “he feels ….” As noted by Russell (1991), there is no clear-cut distinction between emotion and non-emotion words. Some of the labels included in the instrument are rather prototypical emotion labels—including the so-called basic emotion categories—while some might be considered by some scholars as relating to affect rather than emotion or might even be regarded as non-affective words. This, however, should not necessarily be considered as a drawback of this instrument, as it prevents participants to be forced to choose a prototypical emotion label regardless of whether they think the actor is feeling emotional or not. Importantly, besides the 36 labels, participants can also click on either “Neutral/No emotion,” or on “Other”—which activates a text box in which participants are invited to enter their own label in case the instrument does not include the word that came up in their mind while seeing or hearing the actor. Thus, this study implements what can be seen as a semi-forced-choice response format.

The placement of these labels in the two-dimensional space (x, y) has been determined by valence and arousal ratings of words reported in previous research (Whissell, 1989, 2009; Warriner et al., 2013). The mean rating values are used as coordinates for each label in the space (x = valence, y = arousal), determining the position of the labels in the space. The valence values of these labels range from 80 (“hateful”) to 700 (“relaxed”), while the arousal values range from 67 (“tired”) to 764 (“excited”). Some of the values from previous research were slightly adjusted for ease of reading in case two labels would overlap in the space, but adjustments of the values were never <10 units out of 800. It is important to note that the spatial arrangement of the labels was thought up as a visual help for the participants. Since the words are placed in the space according to the valence and arousal levels that people typically assign to them, it is likely that a participant who would have clicked, for instance, somewhere in the upper-right quadrant of the two-dimensional space in phase 1—i.e., indicating a pleasant and agitated feeling—would choose one of the labels typically associated with positive valence and arousal, hence one of the labels placed in the upper-right quadrant. Thus, the participants' response in phase 2 is likely to be faster and less fastidious than if they had to go through a mere hierarchical list of 36 labels. This is known to be an important factor for complete completion of online questionnaires, since participants are likely to drop out before the end or provide low-quality responses—i.e., satisfice—if the task is too long or too fastidious (e.g., Ganassali, 2008). It is crucial to note that participants were explicitly instructed in a tutorial video that this spatial organization does not prevent them to choose a word placed in a different quadrant from the spot chosen in phase 1. An informal exploration of the data confirms that participants did occasionally choose labels that were not necessarily placed in the same quadrant than the spot they chose in phase 1. A pilot study assessing the user-friendliness of the 2DAFS with 6-point Likert-type scales indicated that participants found the spatial organization of the words helpful (“The spatial placing of the words in the square helped me to find the word I wanted to choose,” mean = 3.9, *SD* = 1.3) and that they would not have read the entire set of words if they would have been presented in a list under each other (“I would not read all the words if they were listed under each other instead of placed in the square,” mean = 3.6, *SD* = 1.3). Only a minority of participants would have preferred a list of words rather than this spatial organization (“I would prefer if the words were listed under each other instead of placed in the square,” mean = 2.3, *SD* = 1.3).

### Stimuli

Twelve dynamic stimuli were developed for this study. Originally, stimuli were recorded in the visual–vocal–verbal modality—i.e., with visuals and sound. Next, various versions of the stimuli were created by modulating the available communication modalities, namely, visual–vocal–verbal (audio–visual stimulus), vocal–verbal (audio without visuals), visual-only (visuals without audio), and vocal-only (low-pass-filtered audio recordings making the words indecipherable but retaining prosodic information such as intonation and rhythm). For the present study, stimuli including only visual cues were used. Participants could see a 27-year-old male Mandarin speaker from Beijing but could not hear him. In each of the 10- to 17-s-long recordings, the actor enacts a situation. In accordance with methods used in previous studies (Busso et al., 2008; Volkova et al., 2014; Lorette and Dewaele, 2015), emotion-eliciting scenarios were created for this study. The intended emotions were happy, sad, disgusted, (positively) surprised, afraid, angry, embarrassed, contemptuous, proud, hopeful, and *jiu jié* 纠结—which might be translated to feeling tangled together or in a knot, feeling confusion and chaos due to a difficult situation in which one cannot take a decision—and *wěi qu* 委屈—which might be translated to feeling wronged or feeling unfairly treated. The inclusion of those various intended emotions was motivated by the desire to have emotions which would typically be associated with different levels or arousal and of valence. Note that the purpose of the study was not to investigate the “accurate recognition” of these specific emotions, but to investigate how different emotional states are being perceived by individuals with various cultural backgrounds—regardless of what they were intended to be by the actor. In other words, the focus of this study is not on (dis)agreement between the experiencer and the perceiver, but on interpretation from the perceivers' perspective—i.e., on (dis)agreement between experiencers with different cultural backgrounds. Therefore, various emotions have been included in order to ensure a large array of emotional states that would differ from each other in terms of valence and arousal, especially. For each of the 12 emotional states, a different scenario was imagined, depicting a situation which could typically trigger the emotion in question for a Chinese person. These scenarios were imagined together with two researchers who were born and raised in China in order to guarantee the plausibility of these situations in a Chinese context and avoid a Western bias.

### Participants

The data for this study stems from a bigger dataset where 1,599 participants completed different variants of the same survey—i.e., they were randomly presented with each of the 12 stimuli in one of the four communication modalities investigated in that broader research project (Lorette, 2020). The present study only considers the observations made in the visual-only modality. As the stimulus presentation order as well as the modality of each stimulus was randomized and some participants responded to only a few stimuli before dropping out, different numbers of observations were collected for each stimulus in the visual modality, ranging from 272 to 313. Table 1 presents the demographics for these different observations. Participants come from various countries, with the most represented nationality among non-Chinese participants being Belgian (*n* = 67), French (*n* = 36), British (*n* = 55), American (*n* = 48), Dutch (*n* = 28), and Italian (*n* = 26). Among the Chinese participants, the best represented province was Anhui (*n* = 211), followed by Fujian (*n* = 63) and Jilin (*n* = 63). Although groups differ in terms of age and gender representation, Mann–Whitney U analyses did not reveal any effect of gender on valence and arousal ratings, with all *p* > 0.05, except for one stimulus (*p* < 0.001, difference in location = 79). Regarding age, although Spearman's correlation analyses revealed a significant correlation between age and valence ratings for nine stimuli (0.001 < *p* < 0.039) and between age and arousal ratings for five stimuli, these effects were only weak, with all ρ < 0.2 except for two correlations between age and valence (ρ = 0.25 and ρ = 0.2).

**Table 1:** Demographics of the observations (*n*) for each stimulus.

**Stimulus**	**Culture**	**Gender**				**Total *n***	**Mean age (sd)**
		**Male**	**Female**	**Prefer not to say**	**Other**		
Afraid	Chinese	112	53	1	1	167	22.3 (7.9)
	Non-Chinese	30	90	1	1	122	36.5 (14.1)
	Total	142	143	2	2	289	28.3 (13)
Angry	Chinese	111	74	5	0	190	22.8 (8.8)
	Non-Chinese	27	86	0	1	114	35.4 (12.2)
	Total	138	160	5	1	304	27.5 (11.8)
Contemptuous	Chinese	107	52	1	1	161	21.4 (5.6)
	Non-Chinese	23	88	0	0	111	36.7 (12)
	Total	130	140	1	1	272	27.5 (11.4)
Disgusted	Chinese	111	61	5	0	177	22.1 (6.9)
	Non-Chinese	34	89	3	1	127	37.9 (13.4)
	Total	145	150	8	1	304	28.7 (12.7)
Embarrassed	Chinese	107	67	6	0	180	21.4 (6.1)
	Non-Chinese	30	81	2	0	113	35.9 (12.6)
	Total	137	148	8	0	293	27 (11.5)
Happy	Chinese	109	65	3	1	178	22.3 (8.3)
	Non-Chinese	26	84	1	0	111	37.4 (13.5)
	Total	135	149	4	1	289	28.1 (12.9)
Hopeful	Chinese	109	67	4	0	180	22.4 (8.1)
	Non-Chinese	25	79	1	0	105	34.6 (11.5)
	Total	134	146	5	0	285	26.8 (11.1)
Jiujie	Chinese	110	67	3	2	182	22.5 (8.3)
	Non-Chinese	23	107	1	0	131	34.6 (13.4)
	Total	133	174	4	2	313	27.6 (12.3)
Proud	Chinese	113	66	0	0	179	21.4 (5.5)
	Non-Chinese	30	83	0	0	113	35.3 (13.3)
	Total	143	149	0	0	292	26.8 (11.5)
Sad	Chinese	110	72	3	0	185	22.5 (8.1)
	Non-Chinese	27	84	1	0	112	35.8 (12)
	Total	137	156	4	0	297	27.5 (11.7)
Surprised	Chinese	114	61	4	2	181	22.7 (8.2)
	Non-Chinese	36	89	1	0	126	36.1 (12.7)
	Total	150	150	5	2	307	28.2 (12.5)
Weiqu	Chinese	113	59	5	0	177	21.9 (7.9)
	Non-Chinese	24	82	0	0	106	35.2 (11.9)
	Total	137	141	5	0	283	26.9 (11.5)

### Procedure

The data were collected *via* snowball sampling, with the survey being spread online *via* mailing lists and social media. Participants were invited to click on one of three links provided in the call for participants, depending on the language in which they wished to take the survey, i.e., English, simplified Chinese, or traditional Chinese. The survey and the instrument have been originally developed in English and then translated from English to Chinese by two L1 Mandarin-speaking translators. Translations were reviewed during collaborative discussions until agreement was reached between the translators and were finally reviewed by a third independent translator, with particular attention to concept “equivalence” throughout the whole process in order to minimize ethnocentric biases (Bradby, 2001). Regardless of the version of the survey, the language spoken in the stimuli was Mandarin. The survey could be completed on both desktop and mobile devices, although participants were encouraged to use a desktop device if possible. The emotion perception test was introduced by a short video tutorial—either in Mandarin or in English, depending on the version of the survey—principally aimed at familiarizing participants with the interactive response format. In this tutorial, participants were also introduced to the speaker depicted in the stimuli.

## Results

Due to the presence of heteroscedasticity and departures from normality in the data, Chinese and non-Chinese participants' ratings of valence and arousal were compared *via* Mann–Whitney *U*-tests. Separate analyses were conducted for each stimulus due to the different directions in which an effect could be expected in the various stimuli.

**Figure 3:**
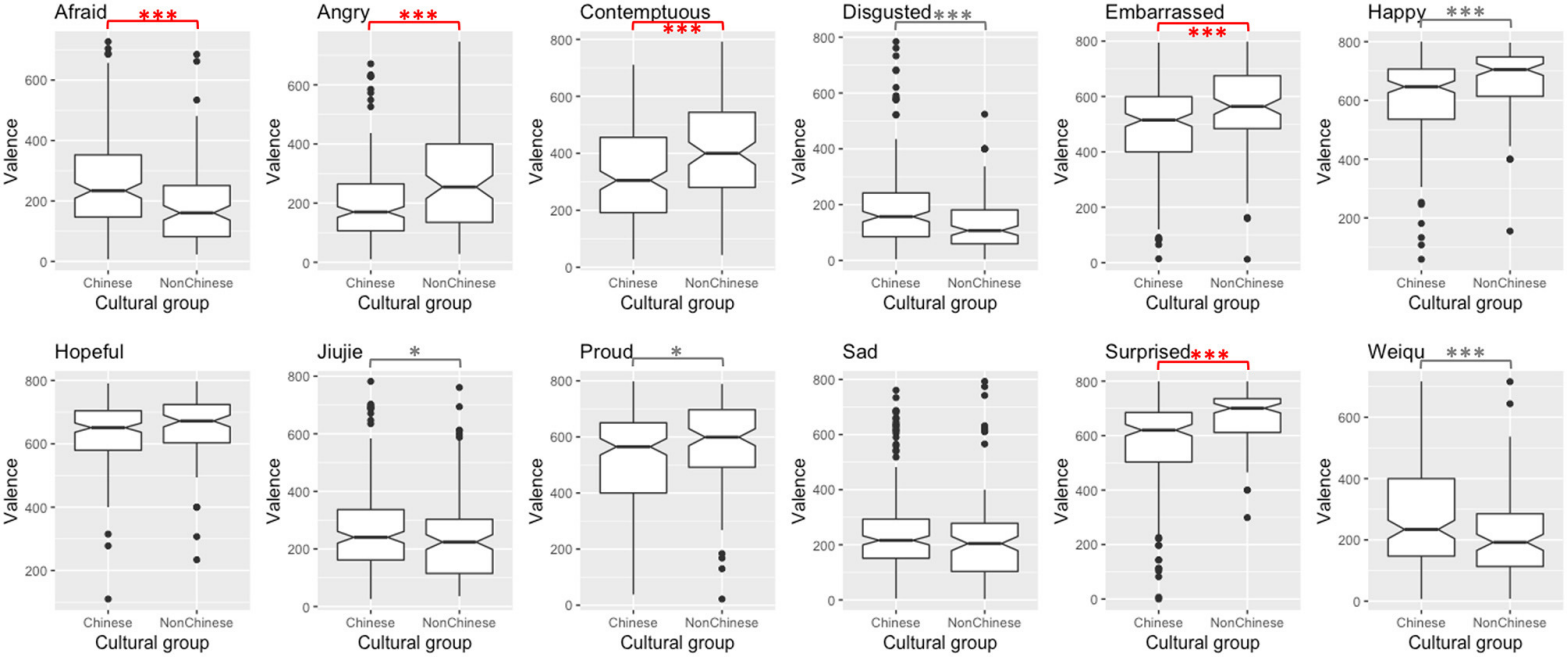
Differences in Chinese and non-Chinese participants' valence ratings per stimulus (***significant at 0.01 level, *significant at 0.05 level, significant and meaningful differences in red, significant and reaching meaningfulness in dotted red, significant but unmeaningful differences in gray).

**Figure 4:**
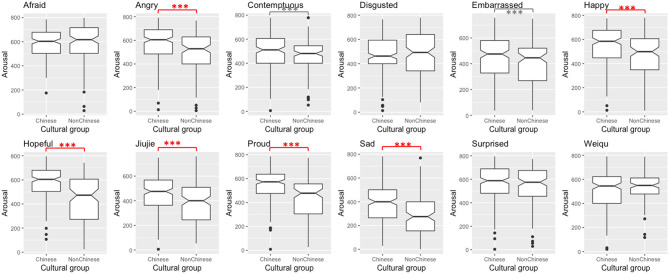
Differences in Chinese and non-Chinese participants' arousal ratings per stimulus (***Significant at 0.01 level, *Significant at 0.05 level, significant and meaningful differences in red, significant and reaching meaningfulness in dotted red, significant but unmeaningful differences in gray.).

Disregarding significant differences smaller than 60—considered meaningless for reasons exposed above, five out of 12 comparisons revealed a significant difference between Chinese and non-Chinese participants' ratings of valence. When there is a difference between Chinese and non-Chinese participants' ratings, non-Chinese participants tend to perceive higher valence levels than Chinese participants—except for one stimulus. For arousal ratings, six out of 12 potential differences turned out significant. In those cases, non-Chinese participants' arousal ratings are lower than Chinese participants' ones. These results are reported in Table 2 and visualized in Figures 3, 4. These findings suggest (slight) cultural variation in valence and arousal perception, although variation was limited and both perceiver groups agreed on the either pleasant or unpleasant and either activated or calm nature of the internal state of the Mandarin speaker. Overall, based on visual cues, non-Chinese participants tend to perceive a Mandarin speaker as feeling more pleasant and less activated than Chinese participants. Thus, this study offers nuanced support to the minimal universality hypothesis (Russell, 2003)—and more generally to the psychological construction approach. This study provides initial evidence in this direction, but future studies based on more representative samples and implementing parametric statistics will need to confirm this trend with more confidence, since the present study is based on snowball sampling, which commonly leads to somewhat unbalanced samples.

**Table 2:** Mann–Whitney *U* analyses for valence and arousal ratings (significant and meaningful differences in bold).

**Stimulus**	**Mean valence ratings**		**Difference in location**	**Mann–Whitney *U***	***p*-values**	**Mean arousal ratings**		**Difference in location**	**Mann–Whitney U**	***p*-values**
	**Chinese**	**Non-Chinese**				**Chinese**	**Non-Chinese**			
Afraid	**267**	**187**	**72**	**13,255**	** <0.001**	582	590	20	9,294.5	0.204
Angry	**200**	**284**	**73**	**7673**	** <0.001**	**572**	**515**	**60**	**13,375**	** <0.001**
Contemptuous	**326**	**415**	**91**	**6,472.5**	** <0.001**	500	459	40	10,330	0.029
Disgusted	198	135	43	14,086	<0.001	463	482	21	10,496	0.325
Embarrassed	**482**	**557**	**71**	**7,584.5**	** <0.001**	450	403	47	11,750	0.026
Happy	610	668	46	7,194	<0.001	**546**	**472**	**77**	**12,478**	** <0.001**
Hopeful	631	649	17	8,445.5	0.135	**582**	**433**	**135**	**13,720**	** <0.001**
Jiujie	270	238	30	13,470	0.05	**455**	**390**	**69**	**14,638**	** <0.001**
Proud	512	567	47	8,434.5	0.017	**554**	**431**	**109**	**14,118**	** <0.001**
Sad	249	225	20	11,353	0.166	**387**	**291**	**101**	**13,758**	** <0.001**
Surprised	**568**	**668**	**72**	**6,537**	** <0.001**	564	537	20	12,356	0.213
Weiqu	270	209	49	11,300	0.004	514	544	18	8,594	0.238

Although it goes beyond the research question investigated in the current study, some raw data obtained in the same sample *via* the second phase of the 2DAFS are presented in Tables 3.1, 3.2 to fully illustrate the possibilities offered by this tool. A descriptive exploration of the data suggests more cross-cultural inconsistency than reported in most previous studies investigating the cross-cultural perception of specific emotions (see e.g., Barrett et al., 2019). It is striking that none of the stimuli yielded an agreement rate above 55%^1^, while proponents of the basic approach have claimed that the so-called basic, universally recognizable emotions “should elicit very high recognition rates, generally in the 70 ± 90% range […] even when methodological constraints are relaxed” (Haidt and Keltner, 1999, p. 238). Instead, agreement rates are in most cases closer to rates observed in free-labeling studies. Although they are still higher than previous free-labeling studies (e.g., Srinivasan and Martinez, 2018; Gendron et al., 2020), those previous studies were based on static stimuli, while stimuli from the present study provide dynamic cues, arguably boosting emotion perception agreement. This reinforces the supposition that, in contrast with traditional forced-choice response formats, the present response format, although slightly more constrained than free-labeling response formats, does not seem to bias responses more than free-labeling response formats.

**Table 3.1:** Frequencies of the label chosen to describe the emotion perceived in each stimulus, with the highest frequency highlighted in blue, frequencies of 10% or more printed in bold, and frequencies of 2% or less printed in gray for ease of reading (105 < *n* <313).

		**Stimulus**											
		**Afraid**		**Angry**		**Contemptuous**		**Disgusted**		**Embarrassed**		**Happy**	
		**Ch**	**NonCh**	**Ch**	**NonCh**	**Ch**	**NonCh**	**Ch**	**NonCh**	**Ch**	**NonCh**	**Ch**	**NonCh**
Chosen label	Afraid	**35 (21%)**	**52 (43%)**	2 (1%)	7 (6%)			8 (5%)	6 (5%)	1 (1%)	1 (1%)	3 (2%)	
	Ambitious	1 (1%)		1 (1%)			2 (2%)				3 (3%)		
	Amorous					2 (1%)		1 (1%)			5 (4%)	1 (1%)	3 (3%)
	Amused					6 (4%)	5 (5%)	1 (1%)		14 (8%)	6 (5%)	**29 (16%)**	8 (7%)
	Angry	5 (3%)		**105 (55%)**	**16 (14%)**	**19 (12%)**	1 (1%)	1 (1%)		1 (1%)			
	Anxious	**27 (16%)**	**33 (27%)**	9 (5%)	6 (5%)	7 (4%)	2 (2%)	**20 (11%)**	**17 (13%)**	4 (2%)	3 (3%)		
	Astonished	**28 (17%)**	4 (3%)	7 (4%)		2 (1%)	2 (2%)				1 (1%)	2 (1%)	4 (4%)
	Bored	2 (1%)			1 (1%)	5 (3%)	1 (1%)	6 (3%)	1 (1%)	4 (2%)		1 (1%)	
	Confident				1 (1%)	1 (1%)	4 (4%)			4 (2%)	6 (5%)	3 (2%)	2 (2%)
	Contemptuous	3 (2%)		2 (1%)	2 (2%)	**37 (23%)**	4 (4%)	1 (1%)	1 (1%)	1 (1%)		1 (1%)	
	Delighted			1 (1%)	2 (2%)	4 (2%)	3 (3%)			13 (7%)	7 (6%)	**24 (13%)**	**17 (15%)**
	Depressed		1 (1%)		1 (1%)	4 (2%)	1 (1%)	**55 (31%)**	9 (7%)	1 (1%)			
	Disappointed	5 (3%)		7 (4%)	**22 (19%)**	9 (6%)	**14 (13%)**	8 (5%)	6 (5%)	2 (1%)	2 (2%)		
	Disgusted	1 (1%)	4 (3%)	2 (1%)	5 (4%)	3 (2%)	4 (4%)	1 (1%)	8 (6%)			1 (1%)	
	Doubtful	1 (1%)	3 (2%)	2 (1%)		1 (1%)	6 (5%)	2 (1%)	2 (2%)	1 (1%)	3 (3%)	2 (1%)	2 (2%)
	Embarrassed	1 (1%)	2 (2%)		5 (4%)	2 (1%)	4 (4%)	2 (1%)	6 (5%)	17 (9%)	6 (5%)	3 (2%)	2 (2%)
	Enthusiastic	1 (1%)			1 (1%)	1 (1%)	4 (4%)			12 (7%)	4 (4%)	6 (3%)	**18 (16%)**
	Excited	1 (1%)		3 (2%)		3 (2%)	2 (2%)			6 (3%)	3 (3%)	**22 (12%)**	**15 (14%)**
	Happy	1 (1%)			1 (1%)					11 (6%)	5 (4%)	**37 (21%)**	**14 (13%)**
	Hateful	2 (1%)		11 (6%)	2 (2%)	5 (3%)	1 (1%)		2 (2%)	1 (1%)			
	Hopeful	1 (1%)			1 (1%)	1 (1%)	10 (9%)	1 (1%)		10 (6%)	**18 (16%)**	7 (4%)	6 (5%)
	Impatient	5 (3%)	1 (1%)	**21 (11%)**	2 (2%)	**24 (15%)**	4 (4%)			2 (1%)	3 (3%)		
	Jealous	1 (1%)	1 (1%)			3 (2%)				2 (1%)			
	Nostalgic	1 (1%)	1 (1%)			1 (1%)	2 (2%)	1 (1%)		4 (2%)	7 (6%)	1 (1%)	2 (2%)
	Proud	1 (1%)			1 (1%)	2 (1%)	2 (2%)			1 (1%)			3 (3%)
	Relaxed	1 (1%)				3 (2%)	3 (3%)			8 (4%)	3 (3%)	6 (3%)	1 (1%)
	Sad				1 (1%)		3 (3%)	**57 (32%)**	**52 (41%)**	1 (1%)	2 (2%)		
	Surprised	15 (9%)	2 (2%)		1 (1%)	2 (1%)	2 (2%)			5 (3%)		3 (2%)	3 (3%)
	Tense	**19 (11%)**	8 (7%)	5 (3%)	3 (3%)	1 (1%)	5 (5%)	1 (1%)	3 (2%)	14 (8%)		3 (2%)	
	Tired					1 (1%)	1 (1%)	1 (1%)		2 (1%)			
	Triumphant									1 (1%)		8 (4%)	5 (5%)
	Solemn			1 (1%)	2 (2%)	1 (1%)			2 (2%)	1 (1%)			
	Dull	1 (1%)	1 (1%)	3 (2%)	2 (2%)					1 (1%)		2 (1%)	
	Relieved					1 (1%)	1 (1%)			8 (4%)	7 (6%)	1 (1%)	1 (1%)
	Serene				1 (1%)	2 (1%)	5 (5%)			5 (3%)	7 (6%)	3 (2%)	1 (1%)
	Mesmerized	1 (1%)			1 (1%)		1 (1%)	1 (1%)		1 (1%)	2 (2%)	4 (2%)	1 (1%)
	Neutral	2 (1%)		3 (2%)	**18 (16%)**	5 (3%)	6 (5%)	2 (1%)		7 (4%)	2 (2%)	1 (1%)	
	OTHER	5 (3%)	9 (7%)	5 (3%)	9 (8%)	3 (2%)	6 (5%)	7 (4%)	12 (9%)	14 (8%)	7 (6%)	4 (2%)	3 (3%)

**Table 3.2:** Frequencies of the label chosen to describe the emotion perceived in each stimulus, with the highest frequency highlighted in blue, frequencies of 10% or more printed in bold, and frequencies of 2% or less printed in gray for ease of reading (105 < *n* < 313).

		**Stimulus**											
		**Hopeful**		**Jiujie**		**Proud**		**Sad**		**Surprised**		**Weiqu**	
		**Ch**	**NonCh**	**Ch**	**NonCh**	**Ch**	**NonCh**	**Ch**	**NonCh**	**Ch**	**NonCh**	**Ch**	**NonCh**
**Chosen label**	Afraid			1 (1%)	1 (1%)				1 (1%)			2 (1%)	3 (3%)
	Ambitious	8 (4%)	3 (3%)			12 (7%)	3 (3%)			2 (1%)	2 (2%)	1 (1%)	
	Amorous	1 (1%)	1 (1%)			2 (1%)		3 (2%)					
	Amused	18 (10%)	6 (6%)	2 (1%)	1 (1%)	**19 (11%)**	**11 (10%)**			**19 (10%)**	9 (7%)	2 (1%)	1 (1%)
	Angry			7 (4%)	3 (2%)	9 (5%)		1 (1%)		2 (1%)		**45 (25%)**	**16 (15%)**
	Anxious	1 (1%)		11 (6%)	7 (5%)		1 (1%)	**31 (17%)**	4 (4%)			14 (8%)	5 (5%)
	Astonished			3 (2%)		5 (3%)		2 (1%)		6 (3%)	7 (6%)	7 (4%)	3 (3%)
	Bored			5 (3%)	5 (4%)	2 (1%)		7 (4%)	3 (3%)	2 (1%)		2 (1%)	
	Confident	9 (5%)	7 (7%)			9 (5%)	6 (5%)	1 (1%)		1 (1%)	2 (2%)	2 (1%)	
	Contemptuous			**24 (13%)**	4 (3%)	7 (4%)		2 (1%)		1 (1%)		14 (8%)	2 (2%)
	Delighted	**33 (18%)**	8 (8%)	4 (2%)		**18 (10%)**	3 (3%)			**24 (13%)**	**12 (10%)**	3 (2%)	
	Depressed			15 (8%)	3 (2%)	1 (1%)		**51 (28%)**	6 (5%)	1 (1%)	1 (1%)	2 (1%)	2 (2%)
	Disappointed	1 (1%)	1 (1%)	**20 (11%)**	**36 (27%)**		3 (3%)	**23 (12%)**	**24 (21%)**	1 (1%)		10 (6%)	**34 (32%)**
	Disgusted			4 (2%)	6 (5%)		5 (4%)	1 (1%)	1 (1%)			3 (2%)	8 (8%)
	Doubtful		3 (3%)	6 (3%)	8 (6%)	1 (1%)	3 (3%)	1 (1%)	**12 (11%)**			2 (1%)	
	Embarrassed			11 (6%)	3 (2%)	1 (1%)	1 (1%)	1 (1%)	6 (5%)			4 (2%)	4 (4%)
	Enthusiastic	15 (8%)	**21 (20%)**		2 (2%)	13 (7%)	**20 (18%)**			11 (6%)	**21 (17%)**	1 (1%)	
	Excited	**19 (11%)**	**15 (14%)**	1 (1%)		13 (7%)	**13 (12%)**			**46 (25%)**	**33 (26%)**	5 (3%)	
	Happy	**24 (13%)**	**10 (10%)**			13 (7%)	5 (4%)			7 (4%)	**15 (12%)**	1 (1%)	
	Hateful			3 (2%)	2 (2%)	4 (2%)	2 (2%)			2 (1%)		4 (2%)	1 (1%)
	Hopeful	15 (8%)	6 (6%)	3 (2%)	2 (2%)	3 (2%)	4 (4%)		1 (1%)	8 (4%)	3 (2%)		
	Impatient		1 (1%)	**19 (10%)**	3 (2%)	7 (4%)	3 (3%)	8 (4%)		3 (2%)		**26 (15%)**	10 (9%)
	Jealous		1 (1%)	1 (1%)	1 (1%)	5 (3%)		1 (1%)		2 (1%)	1 (1%)	5 (3%)	2 (2%)
	Nostalgic	3 (2%)	3 (3%)		3 (2%)		1 (1%)		2 (2%)	1 (1%)			
	Proud	9 (5%)	6 (6%)	2 (1%)		7 (4%)	4 (4%)	1 (1%)		3 (2%)	2 (2%)	1 (1%)	
	Relaxed	7 (4%)	3 (3%)	3 (2%)		3 (2%)	4 (4%)	1 (1%)		3 (2%)		1 (1%)	1 (1%)
	Sad			4 (2%)	**19 (15%)**			16 (9%)	**25 (22%)**				3 (3%)
	Surprised		2 (2%)	5 (3%)	1 (1%)	4 (2%)	7 (6%)	1 (1%)		17 (9%)	11 (9%)	3 (2%)	
	Tense			4 (2%)	6 (5%)	1 (1%)	2 (2%)	9 (5%)	3 (3%)	1 (1%)		6 (3%)	2 (2%)
	Tired			8 (4%)	2 (2%)	1 (1%)		9 (5%)	7 (6%)			1 (1%)	
	Triumphant	6 (3%)	1 (1%)			8 (4%)				5 (3%)	3 (2%)		
	Solemn	1 (1%)			1 (1%)	1 (1%)	1 (1%)		3 (3%)	1 (1%)		1 (1%)	
	Dull			2 (1%)	5 (4%)		1 (1%)		5 (4%)				
	Relieved	2 (1%)		4 (2%)		1 (1%)	3 (3%)			1 (1%)			
	Serene		1 (1%)	1 (1%)		1 (1%)	2 (2%)	2 (1%)	2 (2%)			1 (1%)	
	Mesmerized	3 (2%)	2 (2%)			1 (1%)	1 (1%)	1 (1%)		3 (2%)			1 (1%)
	Neutral	1 (1%)		1 (1%)		2 (1%)	1 (1%)	1 (1%)	1 (1%)	1 (1%)	1 (1%)	3 (2%)	2 (2%)
	Other	4 (2%)	4 (4%)	8 (4%)	7 (5%)	5 (3%)	3 (3%)	11 (6%)	6 (5%)	7 (4%)	3 (2%)	5 (3%)	6 (6%)

## Discussion

The current study provides initial evidence for slight cross-cultural variation in valence and arousal perception, suggesting that the minimal universality hypothesis (Russell, 1995, 2003) might need to be nuanced. These findings were made possible through the 2DAFS, an innovative instrument allowing for both valence and arousal ratings and emotion categorization in big samples. This instrument was developed to respond to the criticism that previous studies into emotion perception lacked ecological validity and predisposed certain findings because their instrument prompted participants to indicate the perceived emotion by choosing from a very restricted list of emotion labels (e.g., Gendron et al., 2014a). The valence and arousal rating phase of the 2DAFS involves a responsive two-dimensional space, based on the experimentally supported assumption that valence and arousal are bipolar dimensions that are correlated (Kuppens et al., 2013) and orthogonal to each other. As reported above, participants found this phase intuitive and user-friendly. The second phase of the 2DAFS, namely, the emotion categorization phase, involves a semi-forced choice that is less constraining than previous response formats. Obviously, this semi-forced choice still constrains the participants' responses to a certain extent. One might argue that free labeling would be a better alternative to forced-choice response format (e.g., Russell, 1994; Gendron et al., 2014b). However, the analysis of free-labeling data from big samples is time-consuming. Most importantly, free labeling arguably merely relegates the categorizing issue—i.e., classifying a potentially infinite number of perceivable emotions into a few categories—from the participant responsibility (in the case of forced-choice response format) to the researcher responsibility (in the case of free labeling). In order to quantitatively analyze such data, the researcher has to cluster the participants' individual responses into broader categories, thus imposing the researcher's own subjective categorization on the participants' responses. Therefore, a semi-forced-choice response format including many proposed emotional labels, a neutral state, as well as a possibility for the participants to enter their own label combines the advantages of forced choice and free labeling, namely, easier analysis of data from large samples and higher ecological validity than strictly forced choice, respectively. However, while providing more labels helps to avoid the limitations linked with very constrained forced-choice tasks, it also introduces issues related to the number of words one is possibly willing to read in an online survey if they are not organized visually, which might in turn introduce some response bias. As explained above, the 38 options were thus organized in space as a visual aid: Labels were placed in the two-dimensional space according to their valence and arousal ratings gathered from previous research (Whissell, 1989, 2009; Warriner et al., 2013). Thus, if participants had indicated very high arousal and very high valence in the first phase of their response, the words that would be the closest to their immediate sightline in the second phase were more likely to be words typically associated with high arousal and high valence. This spatial organization proved to be appreciated by participants, as reported above. In the future, it would be beneficial to confirm participants' impression about the facilitating effect of the special organization of the labels by conducting a comparative study in which half of the participants have to respond *via* the 2DAFS, while the other half has to choose one of the 38 labels from a simple list of words. One could thus compare the response time and the response patterns in both groups to estimate the potential bias introduced by each response format as well as the time needed to respond *via* each response format. Moreover, it would also be interesting to determine whether (and, if so, to what extent) the spatial organization of the labels biases participants' choice patterns. Arguably, participants' label choice in phase 2 might be influenced by their rating in phase 1. However, such a bias is inevitable in any instrument, as a mere hierarchical list of words would also introduce some bias due to the vertical or horizontal organization of the labels. The spatial organization is not meant as a way to completely avoid any response bias, which would be too idealistic of a goal. However, the spatial organization is best regarded as a way to make the instrument less constrained than previous ones and yet more user-friendly and quicker to use, thus limiting participants' fatigue and dropout (Ganassali, 2008).

One limitation of the instrument is that participants can only choose one label per response, thus preventing the report of mixed emotions. Researchers who want to investigate finer-grained perceptions than “the main perceived/experienced emotion” in a stimulus might want to turn to other instruments such as a variant of the GEW enabling more than one response per stimulus [as implemented in Bänziger and Scherer (2010)] or instruments enabling the participants to report the degree to which they experience/perceive each of the proposed emotions (e.g., Ersner-Hershfield et al., 2008; Alqarni and Dewaele, 2018).

To conclude, the 2DAFS is a useful, innovative, and user-friendly instrument that allows for both continuous valence and arousal ratings and categorical emotion perception data in a quick and flexible way. The first phase of this response format, i.e., the core affect rating phase, collects continuous ratings of how (un)pleasant and how (un)activated someone is feeling, enabling the direct assessment of the perception of someone else's (or one's own) core affect. Thus, with the use of this instrument, conclusions on the universal nature of core affect do not need to rely (a) on categorical measurements of valence and/or arousal perceptions which disregard the dimensional conceptualization of valence and arousal (Russell, 2003), or (b) on indirect inferences from response patterns (e.g., Gendron et al., 2018). The second phase of this response format, i.e., the emotion categorization phase, entails a semi-forced choice out of a large list of alternatives. This format allows researchers to overcome the limitations of forced-choice response formats with a very limited number of options—which have been widely used in emotion research but have been shown to bias responses (Gendron et al., 2018). It has the additional benefit of making it less tedious to collect data in big samples than free labeling. Accordingly, the 2DAFS offers an ideal tool that can be easily embedded in online surveys and that can be used by researchers working in different research paradigms, be it in rather categorical or rather dimensional approaches.

## Data Availability Statement

The raw data supporting the conclusions of this article will be made available by the authors, without undue reservation.

## Ethics Statement

The studies involving human participants were reviewed and approved by Birkbeck College School of Science, History and Philosophy. The patients/participants provided their written informed consent to participate in this study.

## Author Contributions

PL has designed the study, has collected, analyzed and interpreted the data, and has written the article on her own.

## Conflict of Interest

The author declares that the research was conducted in the absence of any commercial or financial relationships that could be construed as a potential conflict of interest.

## Footnotes

^1^Note that data from the broader project, involving visual–vocal–verbal cues and thus potentially yielding much higher agreement rates, do not yield agreement rate above 70%.
